# Supplementary material for: Short paired-end reads trump long single-end reads for expression analysis
Source: BMC Bioinformatics. 2020 Apr 19;21:149. doi: 10.1186/s12859-020-3484-z (PMC7168855; doi:10.1186/s12859-020-3484-z)
Supplement: Supplementary file 1 — Additional file 1. Supporting Online Material containing additional supplemental figures and tables. [file 12859_2020_3484_MOESM1_ESM.pdf]

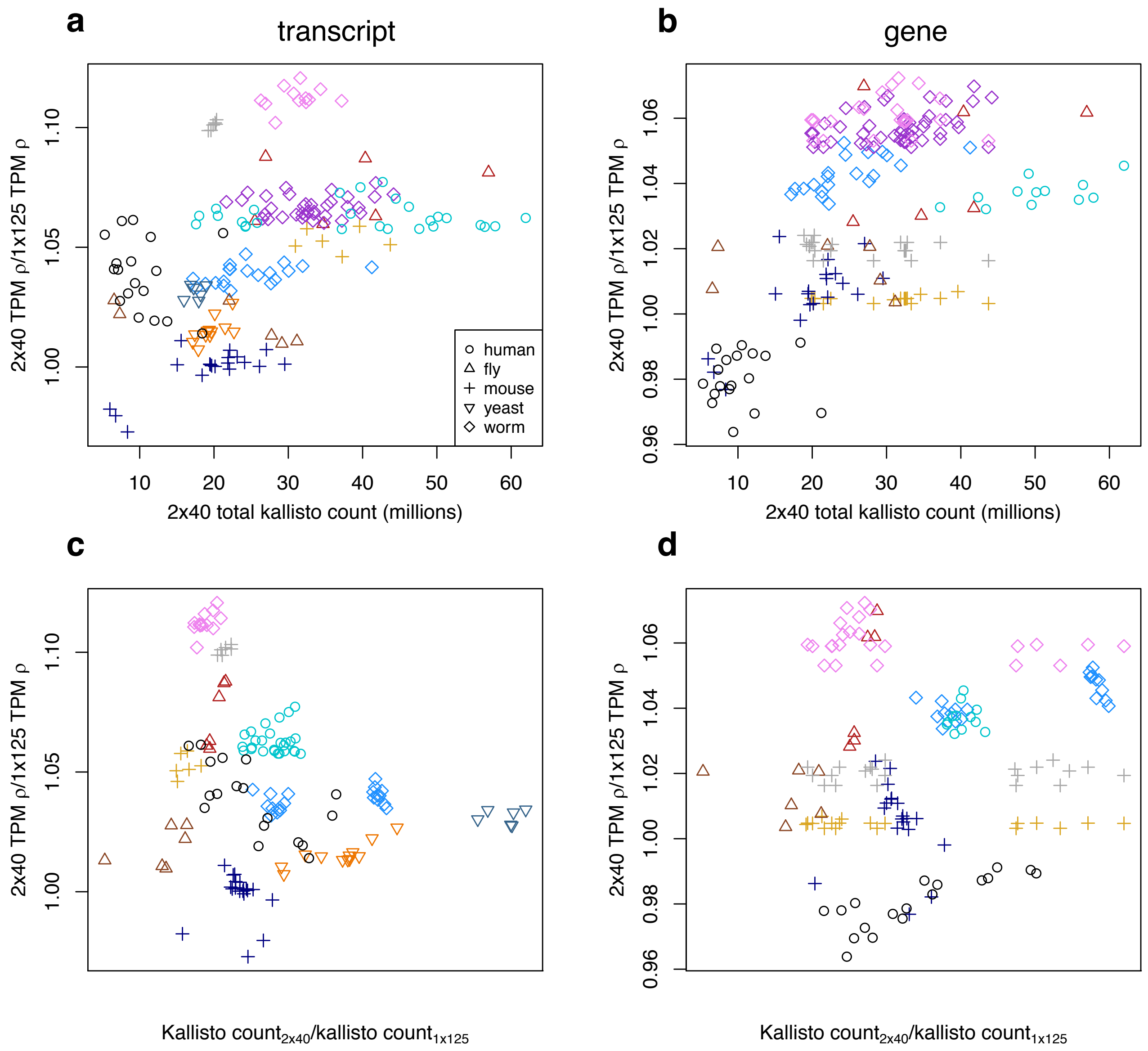

Figure S1. Effects of kallisto pseudo-alignment efficiency on differences in TPM estimate Spearman's rank correlations between 2x40 vs. 1x125. Efficiency of 2x40 is presented as the total kallisto count for 2x40 for **(a)** transcripts and **(b)** genes, and contrasted with the efficiency of 1x125 in the form of count ratios for **(c)** transcripts and **(d)** genes. Symbol colors correspond to SRA accessions.

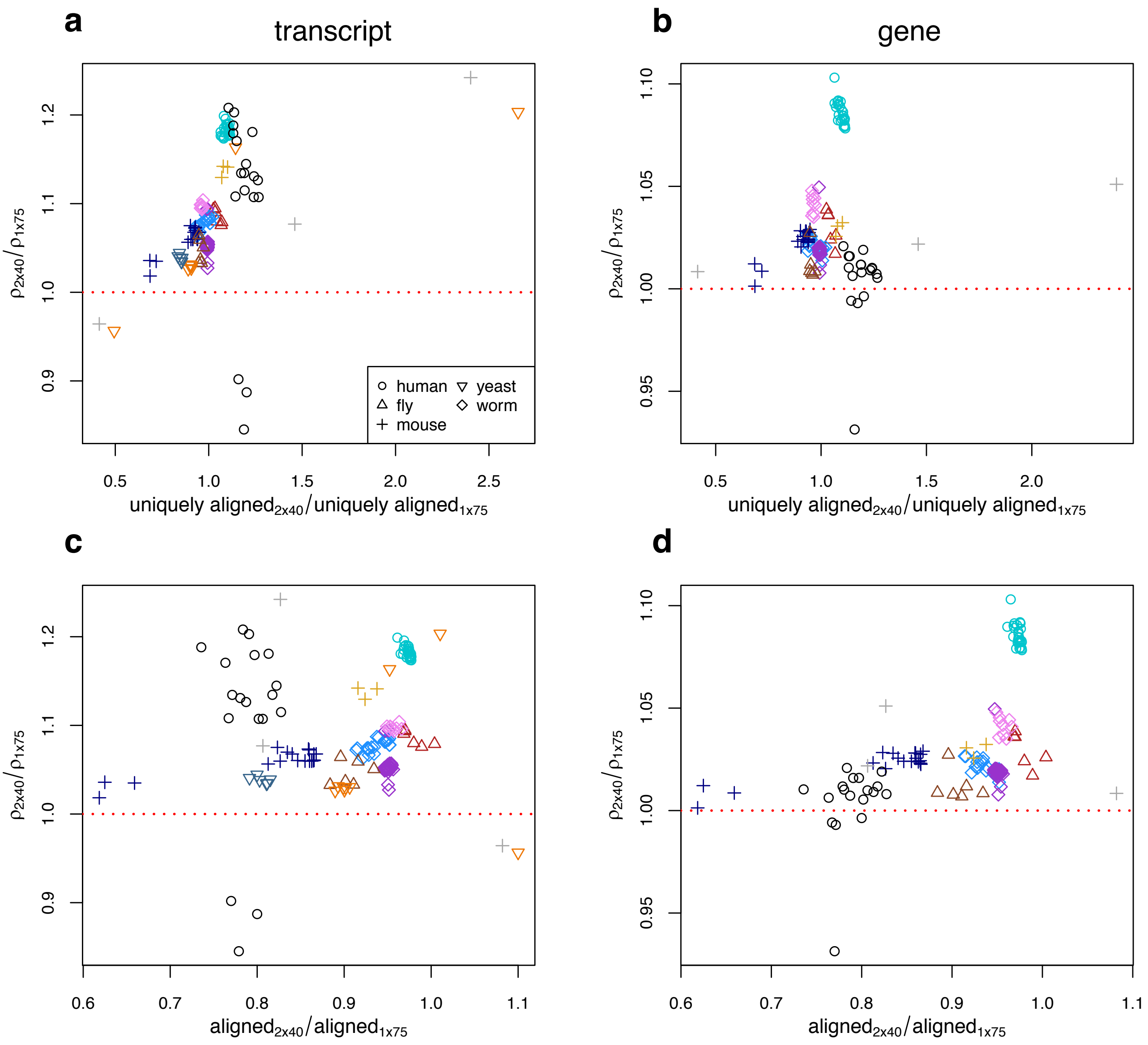

Figure S2. Effects of bowtie2 alignment rates on differences in RSEM TPM estimate Spearman's rank correlations between 2x40 vs. 1x75. Relative differences in efficiency are presented as the ratio of 2x40 to 1x75 TPM correlations with 2x125 TPM over the ratio of unique alignment rate for **(a)** transcripts and **(b)** genes, and over the ratio of overall alignment rates for **(c)** transcripts and **(d)** genes. Symbol colors correspond to SRA accessions.

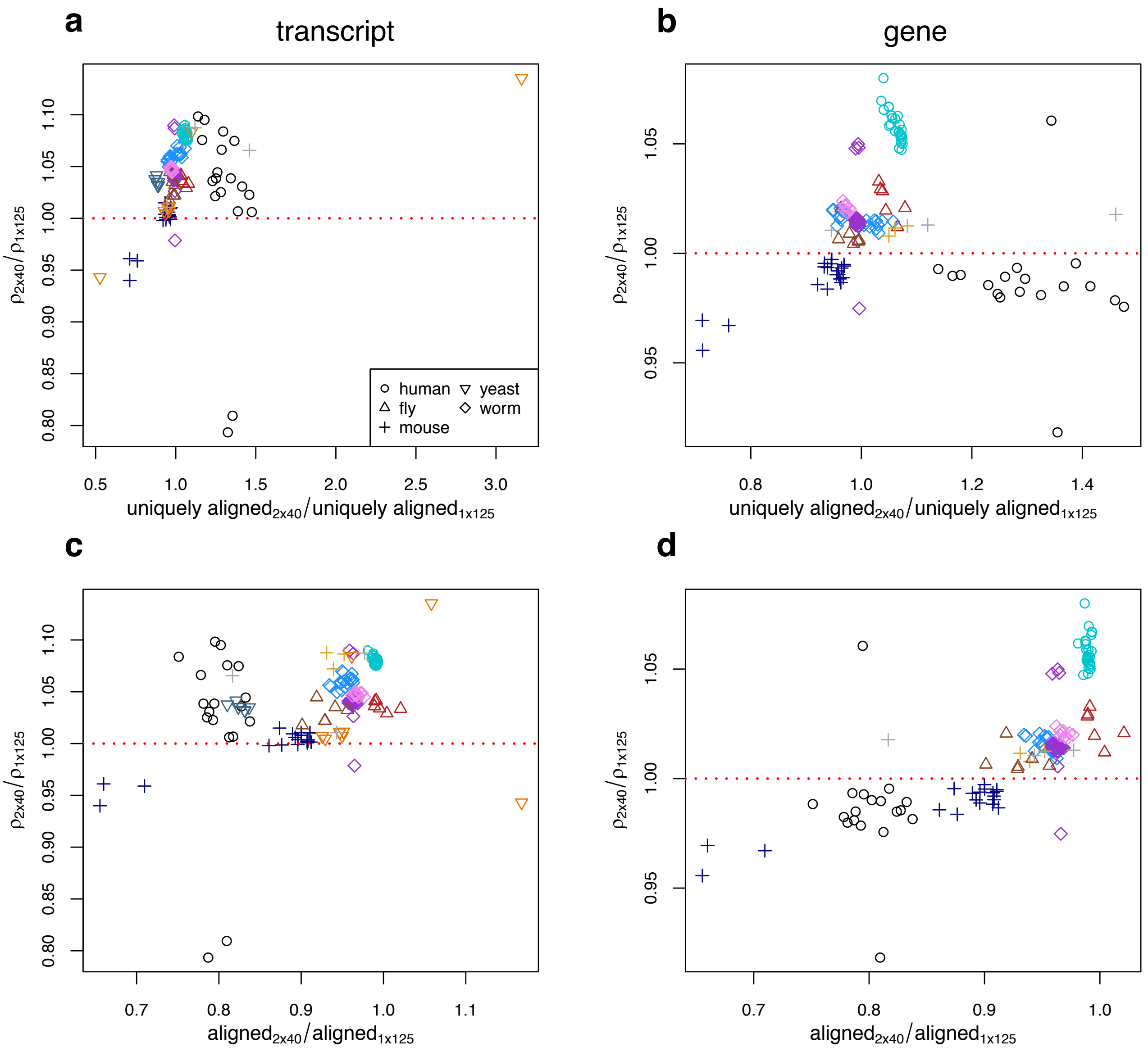

Figure S3. Effects of bowtie2 alignment rates on differences in RSEM TPM estimate Spearman's rank correlations between 2x40 vs. 1x125. Relative differences in efficiency are presented as the ratio of 2x40 to 1x125 TPM correlations with 2x125 TPM over the ratio of unique alignment rate for **(a)** transcripts and **(b)** genes, and over the ratio of overall alignment rates for **(c)** transcripts and **(d)** genes. Symbol colors correspond to SRA accessions.

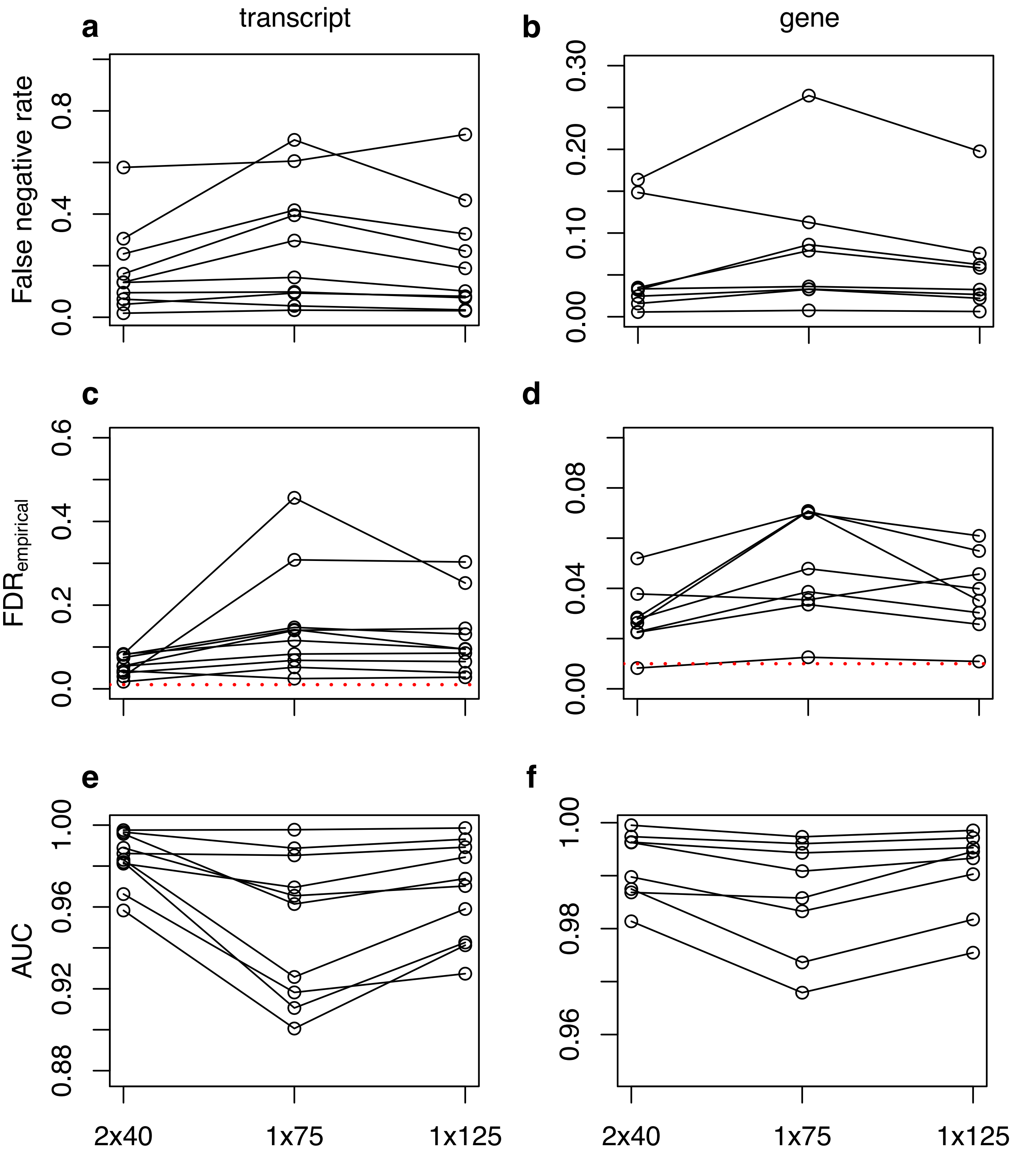

Figure S4. For limma-voom Wald tests, variation across SRA accessions, in **(a,b)** false negative rate**, (c,d)** empirical false discovery rate (red line indicates FDR threshold of 0.01 for calling tests significant), and **(e,f)** AUC between 2x40, 1x75 and 1x125 sequencing strategies. Differences are plotted as means for Wald tests within accessions, for **(a,c,e)** transcripts and **(b,d,f)** genes.

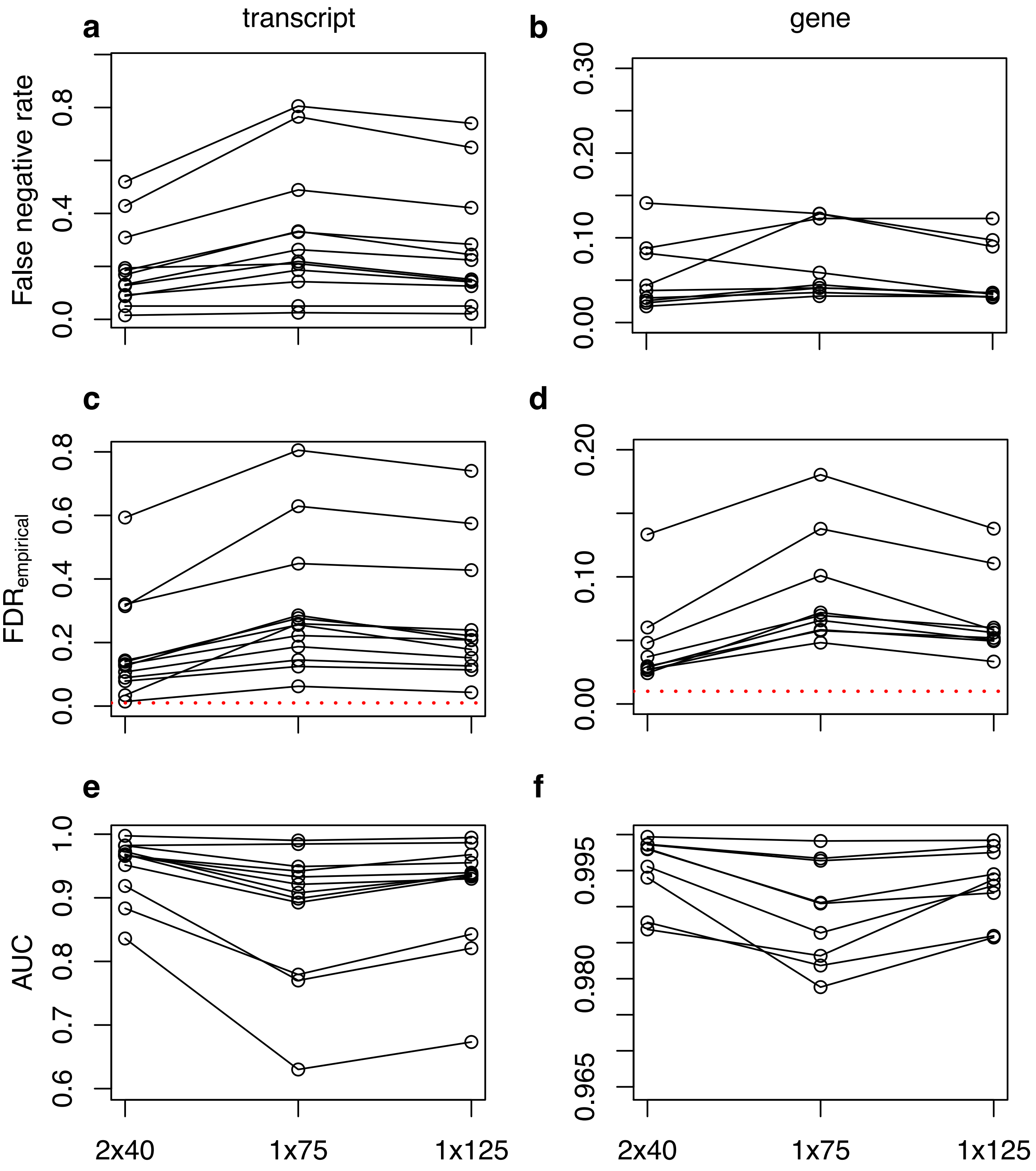

Figure S5. For DESeq2 Wald tests, variation across SRA accessions, in **(a,b)** false negative rate**, (c,d)** empirical false discovery rate (red line indicates FDR threshold of 0.01 for calling tests significant), and **(e,f)** AUC between 2x40, 1x75 and 1x125 sequencing strategies. Differences are plotted as means for Wald tests within accessions, for **(a,c,e)** transcripts and **(b,d,f)** genes.

Table S1. SRA accessions used in this study.

Accession	Organism	Instrument	Paired-end read length	Conditions	Biological replicates per condition	Kallisto	RSEM
SRP133853	*H. sapiens*	HiSeq 2500	125	6	3	x	x
SRP115815	*H. sapiens*	HiSeq 2500	125-126	9	3	x	
SRP105271	*M. musculus*	HiSeq 2000	125	5	3-4	x	
SRP143508	*M. musculus*	HiSeq X Ten	150	2	3	x	
SRP096374	*M. musculus*	HiSeq 4000	150	2	3	x	x
ERP017328	*D. melanogaster*	HiSeq 2500	126	2	3	x	
SRP128516	*D. melanogaster*	HiSeq 4000	151	2	3	x	x
SRP089981	*C. elegans*	NextSeq 500	151	4	5	x	
SRP092256	*C. elegans*	HiSeq 2500	126	7	5	x	
SRP129557	*C. elegans*	HiSeq 3000	126	4	3	x	x
SRP133093	*S. cerevisiae*	HiSeq 2000	151	4	3	x	x
SRP142501	*S. cerevisiae*	HiSeq X Ten	150	2	3	x	

Table S2. Across all assayed SRA accessions, percentages of pairwise differential expression tests between conditions where a performance metric is greater for 2x40 compared to 1x75 or 1x125. Gene-level analyses are not included for yeast accessions (SRP133093 and SRP142501) as this species has no alternative splicing, such that there is only one annotated transcript per gene. Bold values indicate those where 2x40 outperforms the evaluated single-end strategy. NA indicates there were < 50 differentially expressed features such that performance metrics were not calculated.

	transcript		gene	
**Metric**	**2x40 > 1x75**	**2x40 > 1x125**	**2x40 > 1x75**	**2x40 > 1x125**
**sleuth**				
**empirical false discovery rate**				
ERP017328	**0**	**0**	**0**	**0**
SRP089981	**0**	80	**0**	**0**
SRP092256	**0**	**0**	**0**	**0**
SRP096374	**0**	**0**	**0**	**0**
SRP105271	**0**	90	**0**	**0**
SRP115815	**0**	**0**	**0**	**11.1**
SRP129557	**0**	**0**	**0**	**0**
SRP133093	**0**	**0**	**0**	**0**
SRP133853	**46.7**	**46.7**	**46.7**	**46.7**
SRP142501	**0**	**0**	**0**	**0**
**false negative rate**				
ERP017328	**0**	**0**	**0**	**0**
SRP089981	**0**	**20**	**0**	**0**
SRP092256	**0**	**0**	**0**	**0**
SRP096374	**0**	**0**	**0**	**0**
SRP105271	**0**	100	**0**	100
SRP115815	**0**	**8.3**	**0**	**27.8**
SRP129557	**0**	**0**	**0**	**0**
SRP133093	**0**	**0**	–	–
SRP133853	**0**	**0**	**13.3**	**13.3**
SRP142501	**0**	**0**	–	–
**false positive rate**				
ERP017328	**0**	**0**	**0**	**0**
SRP089981	80	80	60	80
SRP092256	**0**	**0**	**0**	**0**
SRP096374	**0**	**0**	**0**	**0**
SRP105271	**0**	90	**20**	80
SRP115815	**0**	**0**	**5.6**	**16.7**
SRP129557	**0**	**0**	**0**	**0**
SRP133093	**0**	**0**	–	–
SRP133853	60	60	53.3	53.3
SRP142501	**0**	**0**	–	–

**sensitivity**				
ERP017328	**100**	**100**	**100**	**100**
SRP089981	**100**	**80**	**100**	**100**
SRP092256	**100**	**100**	**100**	**100**
SRP096374	**100**	**100**	**100**	**100**
SRP105271	**100**	0	**100**	0
SRP115815	**100**	**91.7**	**100**	**72.2**
SRP129557	**100**	**100**	**100**	**100**
SRP133093	**100**	**100**	–	–
SRP133853	**100**	**93.3**	**86.7**	**86.7**
SRP142501	**100**	**100**	–	–
**specificity**				
ERP017328	**100**	**100**	**100**	**100**
SRP089981	20	20	40	20
SRP092256	**100**	**100**	**100**	**100**
SRP096374	0	**100**	**100**	**100**
SRP105271	**100**	10	**80**	20
SRP115815	**100**	**100**	**94.4**	**83.3**
SRP129557	**100**	**100**	**100**	**100**
SRP133093	**100**	**100**	–	–
SRP133853	40	40	46.7	**46.7**
SRP142501	**100**	**100**	–	–
**precision**				
ERP017328	**100**	**100**	**100**	**100**
SRP089981	40	20.0	**100**	40
SRP092256	**100**	**100**	**100**	**100**
SRP096374	**100**	**100**	**100**	**100**
SRP105271	**100**	10	**100**	20
SRP115815	**100**	**100**	**100**	**88.9**
SRP129557	**100**	**100**	**100**	**100**
SRP133093	**100**	**100**	–	–
SRP133853	**53.3**	**53.3**	**53.3**	**53.3**
SRP142501	**100**	**100**	–	–
**auc**				
ERP017328	**100**	**100**	**100**	**100**
SRP089981	**100**	**80**	**100**	**100**
SRP092256	**100**	**100**	**100**	**100**
SRP096374	**100**	**100**	**100**	**100**
SRP105271	**100**	0	**100**	**30**
SRP115815	**100**	**100**	**100**	**100**
SRP129557	**100**	**100**	**100**	**100**
SRP133093	**100**	**100**	–	–

SRP133853	**100**	**100**	**100**	**100**
SRP142501	**100**	**100**	–	–
**limma-voom**				
**empirical false discovery rate**				
ERP017328	**0**	**0**	**0**	**0**
SRP089981	**0**	**0**	**0**	**0**
SRP092256	**0**	**0**	**0**	**5**
SRP096374	100	100	100	**0**
SRP105271	**0**	**0**	**0**	**40**
SRP115815	**0**	**0**	**5.6**	**25**
SRP129557	**0**	**0**	**0**	**0**
SRP133093	**0**	**0**	**0**	**0**
SRP133853	**25**	**25**	**42.9**	50
SRP142501	**0**	**0**	**0**	**0**
**false negative rate**				
ERP017328 (1)	**0**	**0**	**0**	**0**
SRP089981 (4)	**0**	**0**	**0**	**25**
SRP092256 (19)	**21**	89.5	**0**	**30**
SRP096374 (1)	**0**	**0**	**0**	**0**
SRP105271 (9)	**22.2**	77.8	70	100
SRP115815 (36)	**0**	**0**	**0**	**0**
SRP129557 (3)	**0**	**33.3**	**16.7**	**33.3**
SRP133093 (6)	**33.3**	50	–	–
SRP133853 ()???	50	**25**	**35.7**	**42.9**
SRP142501 (1)	100	100	–	–
**false positive rate**				
ERP017328	**0**	**0**	**0**	**0**
SRP089981	**0**	**0**	**0**	**0**
SRP092256	**0**	**0**	**0**	**5.0**
SRP096374	100	100	100	**0**
SRP105271	**0**	**0**	**0**	**20**
SRP115815	**11.1**	**0**	**8.3**	**30.6**
SRP129557	**0**	**0**	**0**	**0**
SRP133093	**0**	**0**	–	–
SRP133853	50	75	57.1	50
SRP142501	**0**	**0**	–	–
**sensitivity**				
ERP017328	**100**	**100**	**100**	**100**
SRP089981	**100**	**100**	**100**	**75**
SRP092256	**78.9**	10.5	**100**	**60**
SRP096374	**100**	**100**	**100**	**100**
SRP105271	**77.8**	11.1	30	0

SRP115815	**100**	**100**	**100**	**100**
SRP129557	**100**	**66.7**	**83.3**	**66.7**
SRP133093	**66.7**	50	–	–
SRP133853	50	**75**	**64.3**	**57.1**
SRP142501	0	0	–	–
**specificity**				
ERP017328	**100**	**100**	**100**	**100**
SRP089981	**100**	**100**	**100**	**100**
SRP092256	**100**	**100**	**100**	**95**
SRP096374	0	0	0	**100**
SRP105271	**100**	**100**	**100**	**80**
SRP115815	**88.9**	**100**	**91.7**	**69.4**
SRP129557	**100**	**100**	**100**	**100**
SRP133093	**100**	**100**	–	–
SRP133853	50	25	42.9	50
SRP142501	**100**	**100**	–	–
**precision**				
ERP017328	**100**	**100**	**100**	**100**
SRP089981	**100**	**100**	**100**	**100**
SRP092256	**100**	**100**	**100**	**95**
SRP096374	0	0	0	**100**
SRP105271	**100**	**100**	**100**	**60**
SRP115815	**100**	**100**	**94.4**	**75**
SRP129557	**100**	**100**	**100**	**100**
SRP133093	**100**	**100**	–	–
SRP133853	**75**	**75**	**57.1**	50
SRP142501	**100**	**100**	–	–
**auc**				
ERP017328	**100**	**100**	**100**	0
SRP089981	**100**	**100**	**100**	**100**
SRP092256	**100**	**100**	**100**	**75**
SRP096374	0	0	**100**	**100**
SRP105271	**100**	33.3	**60**	10
SRP115815	**100**	**100**	**100**	**94.4**
SRP129557	**100**	**100**	**100**	**100**
SRP133093	**100**	**100**	–	–
SRP133853	**100**	**100**	**100**	**71.4**
SRP142501	**100**	0	–	–
**DESeq2**				
**false discovery rate**				
ERP017328	**0**	**0**	**0**	**0**
SRP089981	**0**	**0**	**0**	**20**

SRP092256	**4.8**	**9.5**	**0**	**4.8**
SRP096374	**0**	**0**	**0**	**0**
SRP105271	**0**	**0**	**0**	**20**
SRP115815	**0**	**0**	**2.8**	**8.3**
SRP129557	**0**	**0**	**0**	**0**
SRP133093	**0**	**0**	**0**	**0**
SRP133853	**0**	**0**	**13.3**	**20**
SRP142501	**0**	**0**	**0**	**0**
SRP128516	**0**	**0**	**0**	**0**
SRP143508	**0**	**0**	**0**	**0**
**false negative rate**				
ERP017328	**0**	**0**	**0**	**0**
SRP089981	**0**	**0**	**0**	**0**
SRP092256	**0**	**0**	**9.5**	**42.9**
SRP096374	**0**	**0**	**0**	100
SRP105271	**20**	100	90	100
SRP115815	**0**	**0**	**0**	**0**
SRP129557	**0**	**0**	**0**	**16.7**
SRP133093	**0**	**33.3**	–	–
SRP133853	**0**	**20**	66.7	93.3
SRP142501	**0**	**0**	–	–
SRP128516	**0**	**0**	**0**	**0**
SRP143508	**0**	**0**	NA	NA
**false positive rate**				
ERP017328	**0**	**0**	**0**	**0**
SRP089981	**0**	**0**	**0**	**20**
SRP092256	**9.5**	**14.3**	**0**	**4.8**
SRP096374	**0**	**0**	**0**	**0**
SRP105271	**0**	**0**	**0**	**20**
SRP115815	**0**	**0**	**8.3**	**11.1**
SRP129557	**0**	**16.7**	**0**	**0**
SRP133093	**0**	**0**	–	–
SRP133853	**46.7**	**40**	**20**	**20**
SRP142501	**0**	**0**	–	–
SRP128516	**0**	**0**	**0**	**0**
SRP143508	**0**	**0**	NA	NA
**sensitivity**				
ERP017328	**100**	**100**	**100**	**100**
SRP089981	**100**	**100**	**100**	**100**
SRP092256	**100**	**100**	**90.5**	**52.4**
SRP096374	**100**	**100**	**100**	0
SRP105271	**80**	0	10	0

SRP115815	**100**	**100**	**100**	**100**
SRP129557	**100**	**100**	**100**	**66.7**
SRP133093	**100**	**66.7**	–	–
SRP133853	**100**	**80**	33.3	6.7
SRP142501	0	0	–	–
SRP128516	**100**	**100**	**100**	**100**
SRP143508	**100**	**100**	NA	NA
**specificity**				
ERP017328	**100**	**100**	**100**	**100**
SRP089981	**80**	**100**	**100**	**80**
SRP092256	**90.5**	**85.7**	**100**	**95.2**
SRP096374	**100**	**100**	**100**	**100**
SRP105271	**100**	**100**	**100**	**80**
SRP115815	**100**	**100**	**91.7**	**88.9**
SRP129557	**100**	**83.3**	**100**	**100**
SRP133093	**100**	**100**	–	–
SRP133853	**53.3**	**60**	**80**	**80**
SRP142501	**100**	**100**	–	–
SRP128516	**100**	**100**	**100**	0
SRP143508	**100**	**100**	NA	NA
**precision**				
ERP017328	**100**	**100**	**100**	**100**
SRP089981	**100**	**100**	**100**	**80**
SRP092256	**95.2**	**90.5**	**100**	**95.2**
SRP096374	**100**	**100**	**100**	**100**
SRP105271	**100**	**100**	**100**	**80**
SRP115815	**100**	**100**	**97.2**	**91.7**
SRP129557	**100**	**100**	**100**	**100**
SRP133093	**100**	**100**	–	–
SRP133853	**100**	**100**	**86.7**	**80**
SRP142501	**100**	**100**	–	–
SRP128516	**100**	**100**	**100**	**100**
SRP143508	**100**	**100**	NA	NA
**auc**				
ERP017328	**100**	**100**	**100**	**100**
SRP089981	**100**	**100**	**100**	**100**
SRP092256	**100**	**100**	**100**	**100**
SRP096374	**100**	**100**	**100**	**100**
SRP105271	**100**	40	**90**	0
SRP115815	**100**	**100**	**100**	**100**
SRP129557	**100**	**100**	**100**	**100**
SRP133093	**100**	**100**	–	–

SRP133853	**100**	**100**	**93.3**	**73.3**
SRP142501	0	0	–	–
SRP128516	**100**	**100**	**100**	**100**
SRP143508	**100**	**100**	NA	NA
